# A risk scoring model to predict progression of retinopathy of prematurity for Indonesia

**DOI:** 10.1371/journal.pone.0281284

**Published:** 2023-02-03

**Authors:** Johanes Edy Siswanto, Asri C. Adisasmita, Sudarto Ronoatmodjo, Peter H. Dijk, Arend F. Bos, Florence Manurung, Pieter J. J. Sauer

**Affiliations:** 1 Neonatology Working Group, Department of Pediatrics, Harapan Kita Women and Children Hospital, Jakarta, Indonesia; 2 Faculty of Medicine, Pelita Harapan University, Tangerang, Indonesia; 3 Department of Epidemiology, University of Indonesia School of Public Health, Depok, Indonesia; 4 Department of Pediatrics, Beatrix Children’s Hospital, University Medical Center Groningen, Groningen, The Netherlands; 5 Jakarta Eye Centre, Jakarta, Indonesia; University Medical Centre Ljubljana (UMCL) / Faculty of Medicine, University Ljubljana (FM,UL), SLOVENIA

## Abstract

**Introduction:**

Retinopathy of prematurity (ROP) is a serious eye disease in preterm infants. Generally, the progression of this disease can be detected by screening infants regularly. In case of progression, treatment can be instituted to stop the progression. In Indonesia, however, not all infants are screened because the number of pediatric ophthalmologists trained to screen for ROP and provide treatment is limited. Therefore, other methods are required to identify infants at risk of developing severe ROP.

**Objective:**

To assess a scoring model’s internal and external validity to predict ROP progression in Indonesia.

**Method:**

To develop a scoring model and determine its internal validity, we used data on 98 preterm infants with ROP who had undergone one or more serial eye examinations between 2009 and 2014. For external validation, we analyzed data on 62 infants diagnosed with ROP irrespective of the stage between 2017 and 2020. Patients stemmed from one neonatal unit and three eye clinics in Jakarta, Indonesia.

**Results:**

We identified the duration of oxygen supplementation, gestational age, socio-economic status, place of birth, and oxygen saturation monitor setting as risk factors for developing ROP. We developed two models—one based on the duration of supplemental oxygen and one on the setting of the oxygen saturation monitor. The ROP risk and probabilistic models obtained the same sensitivity and specificity for progression to Type 1 ROP. The agreement, determined with the Kappa statistic, between the ROP risk model’s suitability and the probabilistic model was excellent. The external validity of the ROP risk model showed 100% sensitivity, 73% specificity, 76% positive predictive value, 100% negative predictive value, positive LR +3.7, negative LR 0, 47% pre-test probability, and 77% post-test probability.

**Conclusion:**

The ROP risk scoring model can help to predict which infants with first-stage ROP might show progression to severe ROP and may identify infants who require referral to a pediatric ophthalmologist for treatment.

## Introduction

Retinopathy of prematurity (ROP) is a serious eye disease that can occur in all categories of preterm infants, from extremely preterm to late preterm. The more preterm, the higher the risk of developing ROP. The use of supplemental oxygen is an additional risk factor. Generally, the disease resolves by itself, but it can progress to blindness in some cases [1]. Regular screening of all infants at risk of developing ROP is essential. In most cases, ROP will resolve by itself, but in some cases, it may progress to severe (Type 1) ROP with a risk of blindness. It is important to identify those infants in whom the disease is progressing to Type 1 ROP. Type 1 ROP is defined as ROP that requires treatment. In almost all cases of progressing disease, early intervention can prevent blindness. Screening for ROP is usually performed by ophthalmologists specifically trained to do so. However, trained ophthalmologists are not always present in many hospitals in low and middle-income countries (LMIC), such as Indonesia, that care for preterm infants [2]. It is crucial, therefore, to develop alternative methods or models to identify those infants who are at risk of developing Type 1 ROP.

Although several models have been reported to predict infants at risk of all stages of ROP [3–7], most are unsuitable for LMICs. Our objective was, therefore, to develop other more suitable screening models. Recently, we identified risk factors for the progression of Type 1 ROP in infants in Indonesia [8]. Monitoring these risk factors will help to identify the infants who require regular screening to follow the progression of ROP and those who need treatment. It will help identify those infants who need a transfer to a center where trained ophthalmologists are available [9, 10]. In this paper, we present two models to detect infants at risk to develop Type 1 ROP. One model is based on the setting of the oxygen saturation monitor, while the other is based on the use of supplemental oxygen. We developed two models because oxygen saturation monitors are not available in all hospitals that care for preterm infants. Additionally, we calculated the internal and external validity of our models.

## Methods and statistical analysis

For the study reported here, we used data from our previous case-control study on predicting the progression of ROP [8]. In the paper we included a detailed description of the methodology used. The present study is a retrospective cohort study on the same 98 preterm infants as those who served as cases in our previous case-control study. All 98 infants were diagnosed with ROP and had undergone at least one or more serial eye examinations between 2009 and 2014. We used the data on these 98 cases to develop a scoring model to predict the risk to progress to Type 1 ROP. In addition, a cut-off score was selected by doing a receiver operating characteristics (ROC) analysis, whereby the clinical diagnosis by the ophthalmologists served as the gold standard. The results based on risk scores were then validated using probabilistic models (internal validation).

For external validation, we included 62 infants diagnosed with ROP irrespective of stage and who had undergone one or more serial eye examinations between 2017 and 2020. These infants were recruited from the neonatal intensive care unit of Harapan Kita Women and Children Hospital and three eye clinics in Greater Jakarta. All preterm infants with gestational age (GA) of less than 34 weeks or a birth weight of less than 1500 g, who were diagnosed with ROP, were included in this study. We also included preterm infants born after 34 weeks if they showed cardiorespiratory instability during the first days after birth and required supplemental oxygen.

The study was approved by the Institutional Review Board of the Harapan Kita National Center for Women and Children’s Health. The IRB waived the need for Informed consent because all data collected came from medical records and all data were anonymized. The Kappa statistic was calculated to validate the reliability of the scoring model. Finally, we designed a flow chart for use by the ophthalmologist or assisting physician to identify the infants at risk of progressing ROP.

### Designing the models for progressing ROP

The initial step taken to design a risk scoring model is to identify risk factors for the progression of ROP in infants in Indonesia [8]. These risk factors formed the basis for the models we described the relationship between the dichotomous characteristics, Type 1 ROP or not Type 1 ROP, as outcome and risk factors as a set of independent variables. We used multivariate analysis, using multiple logistic regression, to create clinical prediction models to estimate the probability that an individual patient is at risk of progressing ROP. The diagnostic model developed to assist in establishing a diagnosis for each patient can be presented in several ways. The most appropriate method is to report the original (unmodified) model with a shrinking regression coefficient and appropriate model discrimination. The presentation of this model is in the form of an equation:

***Log [probability (outcome event)/probability (non event)] = β***_***0***_ ***+ β***_1_*T_***1***_ ***+ β***_2_*T_***2***_ ***+ …… β***_n_*T_***n*,**_ in which β_0_ is the intercept and β_1_ to βn are regression coefficients of T_1_ to T_n_. T_1_ to T_n_ are the results of the diagnostic determinants (risk factors). We called this equation the probabilistic model. The next step was to estimate the diagnostic accuracy of this reduced (shrunken) multivariate model. Discrimination commonly estimates the accuracy of a model. The discrimination of a multivariate model refers to the model’s ability to discriminate between subjects with and without the disease. This is estimated with the area under the ROC curve [10].

Using this multiple logistic regression equation models in daily practice proved difficult. This led us to design a simpler model to predict the probability of progression towards Type 1 ROP. A simplified risk score or scoring rule is an adequate alternative to present a prediction model and may facilitate its implementation. We transformed the original (shrunken) regression coefficients of the first method to rounded numbers that can be added up easily. This is commonly done by dividing each regression coefficient by the smallest regression coefficient, multiplying by 10, and rounding to the nearest integer [10, 11]. We used the β coefficient from the multivariate analysis to make a predictive scoring model for ROP. The steps were as follows: Step 1, multiply β by 10; Step 2, divide the results of the first step by the smallest number from Step 1. Conversion to a scoring system to facilitate calculation is done by using the following formula:

$$\frac{\beta \;coefficient\;x\;10}{The\;results\;of\;the\;smallest\;\beta \;x\;10}$$

The smallest number obtained from the quotient is used as a reference for determining the risk score against the outcome. We used the risk factors generated from previous studies to assess the probability of the occurrence of Type 1 ROP. The *P-value* and relative risk of factors related to the progression of the ROP are shown in Table 1. Although simplification of the model allows for loss of information in diagnostic accuracy, it usually does not affect clinical relevance. This ROP risk model is expected to be applied with ease at various levels of high-risk perinatal services.

**Table 1:** Modified calculation of scores for the establishment of an ROP risk model.

Score model	Variable	*P*	ARR (95% CI)	β	β x 10 rounded	β /smallest number	Scoring Model A	Scoring Model B
calculation								
**Model A (FiO**_**2**_ **model)**	**Oxygen supplementation**							
	>7 days	0.051	4.16 (0.99–17.41)	2.26	23	4.6	4.6	
	1–7 days	0.604	1.54 (0.30–7.95)	0.54	***5***	1	1	
	Without O2	-	1.00	-	-	-	-	
	**Socioeconomic status**							
	Lower	0.021	0.36 (0.15–0.86)	-1.76	-18	-3.6	-3.6	
	Middle–upper	-	1.00	-	-	-	-	
**Model B (SpO**_**2**_ **model)**	**Gestational age**							
	<28 weeks	0.007	2.79 (1.33–5.83)	2.77	28	3.1		3.1
	≥28 weeks	-	1.00	-	-	-		-
	**Patient access**							
	Outborn infants	0.017	2.32 (1.16–4.65)	1.58	16	1.8		1.8
	Inborn infants	-	1.00	-	-	-		-
	**Lowest SpO**_**2**_							
	≥93.50%	0.018	2.83 (1.19–6.74)	2.63	26	2.9		2.9
	85.00–93.5%	0.203	1.70 (0.75–3.83)	0.88	***9***	1		1
	<85%	-	1.00	-	-	-		-

We further divided each model into two parts to calculating the probability of the risk of Type 1 ROP. One is based on the mode and duration of supplemental oxygen (Model A). The other is based on the setting of the oxygen saturation monitor (Model B). Our models are based on a survey of infants with ROP from four neonatal intensive care centers and three eye clinics in Jakarta, Indonesia, between 2009 and 2014, and diagnosed with any stage of ROP by experienced pediatric ophthalmologists [8].

### Internal and external validation

To test and compare the performance of the probabilistic and risk scoring models, with the ophthalmologic examination as the gold standard, we carried out an internal validation using the 98 cases from the previous study (8). We carried out an external validation using the 62 new cases. We determined validity on the basis of the area under the curve (AUC) discrimination value on the ROC chart. The AUC is a combination of sensitivity and specificity that reflects the quality of the diagnostic test. The AUC result can be used to assess and predict the progression of ROP.

External validation using new data was required before the model can be used with confidence in clinical practice [12, 13]. External validation involved testing the model in new patients. The term external refers to the use of data from subjects who were not included in the study in which the prediction model had been developed [10].

Another way of validating the use of the ROP risk model is to compare the Kappa value between the risk scoring model and the probabilistic model in assessing the probability of progression to Type 1 ROP. The Kappa statistic validates the scoring model’s reliability; a good result will have a value close to 1 (range 0–1). We carried out this test because there are differences in the calculation method between the risk scoring and probabilistic models. Kappa statistics are often used to test the reliability between assessments. The importance of reliability assessment lies in the fact that data or results collected in the study accurately represent the variables being measured. The extent to which data collection assessments give the same score for the same variable is referred to as the inter-reliability assessment. Cohen’s Kappa results are interpreted as follows: values ≤0 indicate no agreement, between 0.01–0.20 indicate no to slight agreement, 0.21–0.40 fair agreement, 0.41–0.60 moderate agreement, 0.61–0.80 substantial agreement, and 0.81–1.00 indicate almost perfect agreement [14]. Statistical analysis was done using the statistical package for social science (IBM SPSS Statistics for Windows, Version 24.0. Armonk, NY: IBM Corp).

### Pre-test and post-test probability

Pre-test probability is the probability of a condition being present before a diagnostic test is performed. Post-test probability is the probability of a condition being present after a diagnostic test. The estimated pre-test probability of disease can be combined with likelihood ratios. These ratios have several strong properties that make them more useful clinically compared to other accuracy measures in provide an estimate of the post-test probability of disease. Pre-test probabilities of disease are converted to pre-test odds of disease. It is then multiplied by the likelihood ratio to give the post-test odds of disease, transformed into the post-test probability of disease. The calculation is done using a mathematical relationship known as Bayes’ theorem. Alternatively, a tool known as a Fagan nomogram can be used to derive the post-test probability of disease estimates for any combination of disease pre-test probabilities and likelihood ratios [15].

## Results

### Designing the models for progressing to Type 1 ROP

Using the techniques described above, we developed a score based on the probabilistic model as well as a modified risk score. This was done for both models using the oxygen given (Model A: FiO_2_ model) and the setting of the saturation monitor (Model B: SpO_2_). We used the same five risk factors for the ROP risk scoring model. The modified calculation of the risk score for progressing ROP is shown in Table 1.

The probabilistic score for Model A is: Logit (Y | Type 1 ROP) = duration_O_2__>7 days * 1.42 + duration_O_2__1-7days * 0.43—SES Low * 1.03 (*P* value = 0.002), while for Model B: Logit (Y | Type 1 ROP) = GA <28weeks * 1.02 + outborn * 2.32—lowest _SpO_2__≥93.5% * 1.04 + lowest_SpO_2_%_85–93.5% * 0.53 (*P* value = 0.009).

#### Comparison and calculation of internal validity between scoring and probabilistic models

Table 2 shows the comparison of the validity between the risk scoring model and the probabilistic model, with clinical diagnosis as the gold standard. The sensitivity and specificity results are the same for both models. The discrimination ability of a score is given by the area under the ROC curve (AUC). A value above 0.7 indicates that the score may be useful in practice, while a value of 0.8 or more indicates that the score is good [16, 17].

**Table 2:** Testing the validity of the risk scoring model and the probabilistic model for the risk of developing ROP with clinical diagnosis as the gold standard.

ROP diagnostic model		Clinical diagnosis (gold standard)		
		AUC (confidence Interval)	Se	Sp
Risk scoring model	Model A (FiO_2_)	0.751 (0.653–0.849)	65%	79%
	Model B (SpO_2_)	0.804 (0.707–0.902)	80%	71%
Probabilistic model	Model A (FiO_2_)	0.751 (0.653–0.849)	65%	79%
	Model B (SpO_2_)	0.804 (0.707–0.902)	80%	71%

The AUC for Model B was more than 0.8 and thus slightly higher than that for Model A, indicating a good discriminating ability. There was almost no difference between a probabilistic model’s score and an adjustable risk score. In order to confirm the good correlation between the probabilistic model and the risk score, we calculated the Kappa statistic and found a value of 1,000, which indicates excellent agreement. The advantage of the risk scoring model lies in its applicability in daily practice. Calculating the risk model for ROP can be done manually in the neonatal care unit by all health workers by simply adding up the scores of each risk factor present. The final model for the ROP risk model is shown in Table 3. The cut-off point for Model A is 2.8, and for Model B, it is 0.9. Type 1 ROP risk is present when the calculation result exceeds the cut-off value.

**Table 3:** The ROP risk scoring model.

Score model calculation	Variable	(a)	(b)	y(n) = (a) x (b)	Score	∑ scoring model
		Constant	If Yes = 1, if No = 0			
**Model A (FiO**_**2**_ **model)**	**Oxygen supplementation**					**Model A** (y1+y2)
	>7 days	4.6			y1	
	1–7 days	1				
	Without O_2_	0				
	**Socioeconomic status**					
	Low	-3.6			y2	
	Middle–upper	0				
**Model B (SpO**_**2**_ **model)**	**Gestational age**					**Model B** (y3+y4+y5)
	<28 weeks	3.1			y3	
	≥28 weeks	0				
	**Patient access**					
	Outborn infants	1.8			y4	
	Inborn infants	0				
	**Lowest SpO**_**2**_					
	≥93.5%	2.9			y5	
	85–93.5%	1				
	<85%	0				

Note:

Scoring Model A Risk of Type 1 ROP if the sum of scores ≥ 2,8

Scoring Model B Risk of Type 1 ROP if the sum of scores ≥ 0,9

Patients have an increased risk of Type 1 ROP (progression of ROP) if the resulting total score meets or exceeds one of the cut-off values

### External validation of the risk score

Sixty-two infants were included, GA 25–37 weeks, birth weight 600–2000 g. Fifty infants had a GA ≤ 32 weeks, 13 a GA <28 weeks. Twenty-nine infants had Type 1 ROP, and 33 infants had Type 2 ROP (group classification based on the final results of the early treatment for retinopathy of prematurity (ETROP) protocol 2004 [17]. Based on the International Committee for the Classification of Retinopathy of Prematurity (ICROP) revisited 2005 grouping, 27 infants had Stage 1–2, and the remaining 35 infants were Stage 3–5 [18]. In Table 4, infants are divided according to the type of ROP and place of birth.

**Table 4:** Characteristics of 62 cases of ROP by type of ROP and place of birth.

Variable	Outborn infants			Inborn Infants			
	GA <28 wks	GA 28–32 wks	GA >32 wks	GA <28 wks	GA 28–32 wks	GA >32 wks	Total
Type 1 ROP	6	13	1	2	6	1	29
Type 2 ROP	2	6	2	3	12	8	33
Total	8	19	3	5	18	9	62

Abbreviations: ROP—Retinopathy of prematurity; GA—gestational age; wks—weeks.

Note: The weight of the heaviest infant with GA >32 weeks who reached Type 1 was 1806 grams. The highest weight of an infant treated for ROP was 1350 grams.

In the note of Table 3, we explain how to predict Type 1 ROP and differentiate it from Type 2. The calculation of cut-off values of Models A and B each stands alone with probability scores that are also different between the two models (A > = 2.8; B > = 0.9). Put differently, the score of Type 2 Model A <2.8 and Model B <0.9. For the benefit of our readers, we present a 2 x 2 table of calculation results based on predicted scores of Model A (FiO_2_), Model B (SpO_2_), and the combination model of Models A and B compared to gold-standard clinical diagnoses by ophthalmologists (Table 5).

**Table 5:** The 2 x 2 table to calculate the diagnostic test of the scoring system against the gold standard clinical diagnosis.

Diagnosis ROP		Clinical diagnosis by ophthalmologist		
		Type 1 ROP	Type 2 ROP	Total
Diagnosis based on scores from predictive models	Model A (FiO_2_)			
	Risk score Type 1 ROP	26 cases	24 cases	50 cases
	Risk score Type 2 ROP	3 cases	9 cases	12 cases
		29 cases	33 cases	62 cases
	Model B (SpO_2_)			
	Risk score Type 1 ROP	22 cases	13 cases	35 cases
	Risk score Type 2 ROP	7 cases	20 cases	27 cases
		29 cases	33 cases	62 cases
	Combined Models A and B			
	Risk score Type 1 ROP	29 cases	9 cases	38 cases
	Risk score Type 2 ROP	0 cases	24 cases	24 cases
		29 cases	33 cases	62 cases

The results of the calculation of the external validation of the risk score is shown in Table 6. The diagnostic test of the combination of the two models is used as a basis for considering the application of the risk factor model in daily practice in referring infants with a high-risk infants for Type 1 ROP to an adequate eye care facility. After precise calculation of the diagnostic test, the sensitivity of Model A is 90%, the positive predictive value 52%, and the negative predictive value 75%. The results for Model A are somewhat higher than for Model B. The sensitivity is 100% when both models are combined, the NPV 100%.

**Table 6:** External validity of Type 1 ROP based on the ROP risk scoring model.

Study evidence	Prediction of Model A (FiO_2_)	Prediction of Model B (SpO_2_)	Predictions of combined Models A and/or B
Sensitivity	90	76	100
Specificity	27	61	73
PPV	52	63	76
NPV	75	74	100
LR +	1.2	1.9	3.7
LR -	0.4	0.4	0

Abbreviations: PPV—positive predictive value; NPV—negative predictive value; LR +—positive likelihood ratio; LR—- negative likelihood ratio

### The pre-test and post-test probabilities

**Fig 1:**
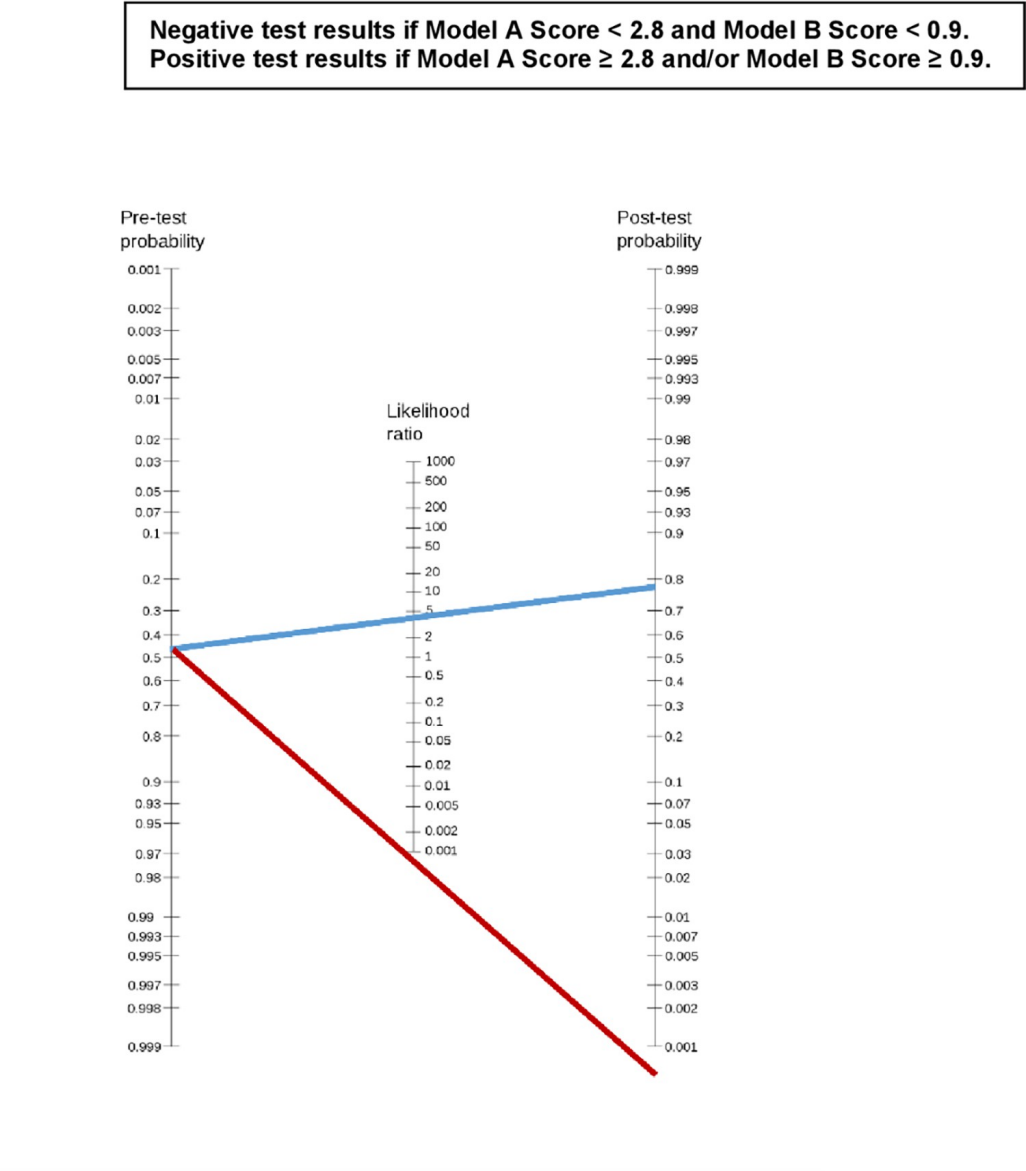
Pre-test and post-test probabilities of the risk scoring model. Calculation of post-test probabilities using likelihood ratio. Pre-test probability = p = (TP+FN)/(TP +FP + FN +TN) = 0.47. Pre-test odd = p/1-p = 0.47/0.53 = 0.89. Post-test odds = pre-test odds x likelihood ratio. Post-test odds = o = 0.89 x 3.7 = 3.3. Post-test probability = o/(1 +o) = 3.3/4.3 = 0.77.

An illustration of Fagan’s nomogram to calculate the pre-test and post-test probabilities using the combined risk score models can be seen in Fig 1. The pre-test probability calculation result in this case was 0.47 (28 cases of Type 1 ROP among 62 infants with ROP). If an infant’s test (risk score) result is positive, the post-test probability of this case will be 0.77. This figure shows a relatively high trend of ROP progression. On the contrary, if the test result is negative, the post-test probability of this infant would be 0.00, meaning that there is no risk of progression to Type 1 ROP.

## Discussion

The increasing number of intensive care units for preterm and sick full-term infants in LMICs such as Indonesia has led to an increase in the survival of preterm infants. This higher survival rate also means increasing numbers of infants with ROP. In Indonesia, national screening and therapy guidelines for ROP were introduced in 2009. However, these guidelines were not implemented as well as they could have been because of the limited number of trained ophthalmologists and financial constraints. This situation results in an increasing number of ROP cases, including blindness in some cases, which could have been prevented if proper screening and treatment had been available.

Studies have shown that 90% of infants with ROP experience regression of the disease. Nevertheless, if left untreated, 10% of the infants suffer from the progression of ROP into Type 1, which can result in severe eye damage or even blindness [1]. It is estimated that 10% to15% of infants in LMIC are born preterm, compared to 7% in high-income countries [19, 20]. Methods to detect infants at risk of progressing ROP is, therefore, of the utmost importance. In a recent Indonesian survey, we found that the incidence of Type 1 ROP in teaching hospitals in infants born <28 weeks was 16 to 22%, and 10% in infants born after 28–32 weeks [2]. These numbers are much higher compared to high-income countries.

New strategies are needed that offer more suitable solutions for rapid diagnosis and timely treatment of ROP. What is required is rapid remote diagnoses by experienced ophthalmologists and personnel trained in retinal imaging using camera technology at an affordable price and adequate capacity. Especially when taking into consideration the vast area that must be served and the geographic difficulties that must be overcome in an extensive archipelago like Indonesia. The above requirements could be combined with an ROP scoring model to identify those infants who run a high risk to develop Type 1 ROP, which may progress to severe sequelae like blindness. Timely referral of high-risk infants to a center where the expertise is available to diagnose and treat ROP may prevent the progression of ROP.

A number of ROP scoring models have been published during the past ten years. The first was described by Hellstrom based on early weight gain and the insulin-like growth factor 1 (IGF-1) level [3]. The same group published an article stating that a model based only on early weight gain could predict the development of all stage ROP because weight gain is closely related to the (IGF-1) level [4]. Binenbaum stated that a model based on birth weight, gestational age, and weight gain could predict the development of Type 1 ROP with a sensitivity of 98.5% in infants with a birth weight of less than 1500 g and gestational age of less than 30 weeks [5]. Eckert and colleagues described a model based on birth weight, gestational age, blood transfusion, the need for mechanical ventilation, and weight at six weeks postnatal age. In that study, ROP of any stage was found in 23% of the infants, severe ROP in 5%. The area under the ROC curve for all stages of ROP was 0.77 and for severe ROP was 0.88 [6]. The Colorado Retinopathy of Prematurity (CO-ROP) found a sensitivity of all stages of ROP of 96.9% and a specificity of 40.9% in a model based on birth weight, gestational age, and weight gain from birth to 28 days. Three percent of infants with severe ROP were missed using that model [7].

The sensitivity of our models to detect infants at risk of developing severe ROP is in line with the above studies, despite the fact that our infants had a higher gestational age and birth weight compared with the studies of Eckert and Binenbaum. In our population, the use of oxygen was the most important risk factor in developing progressive ROP, as well as in infants with a higher gestational age and birth weight. Birth weight, place of birth, and socioeconomic factors also influenced the risk. We speculate that the use of oxygen is not controlled in our population in the same way as in high-income countries where oxygen blenders and saturation monitors are widely available. Therefore, oxygen is still an important risk factor in Indonesia.

Nevertheless, in contrast to studies from Brazil (2012), Spain (2020), and China (2021) that only emphasize the duration of oxygen use with mechanical ventilation [6, 21, 22], in our study, we added the variable ’low setting saturation monitor’ which is most meaningful in terms of ROP progression. We did not find that weight gain was a predictive factor. This might be due to the rather low weight gain in most of our infants on account of insufficient–parenteral—nutrition and the slow progression of enteral feeds due to the frequent presence of complications like sepsis.

We traced the common thread of the relationship between low socio-economic factors, accessibility to oxygen, and the development of ROP. According to our model, a low socio-economic condition prevents the development of severe ROP due to a higher mortality in this group. Previous studies showed that a high mortality rate due to a low target oxygen saturation was related to a lower incidence of ROP [23, 24]. Lower target range of oxygen saturation are advised to prevent reactive oxygen species (ROS)—related diseases, such as retinopathy of prematurity (ROP) and bronchopulmonary dysplasia (BPD) [24]. Until recently, the options to be treated and survive were limited for preterm infants from low socio-economic backgrounds in Indonesia. The chance that these infants received supplemental oxygen was lower than that of infants from families with good health care insurance. Survival rates in these infants were also lower. Put differently, infants from families with good health insurance had a higher chance of receiving supplemental oxygen and surviving and hence a higher risk to develop ROP. Infants from a low socio-economic condition that survived were most likely less sick and received less oxygen compared to surviving infants from families with good insurance. We found that outborn infants had a higher risk to develop Type 1 ROP. Outborn infants need to be transported from a local hospital to the neonatal center. This transport is done with an ambulance, where oxygen is available, but no saturation monitor and oxygen blender. During this transport, the chance of giving a high amount of oxygen is likely. A high amount of oxygen is given to prevent complications during transport. The oxygen given increases the chance to develop ROP.

Diagnostic tests are more useful when the post-test probability shows a significant change than the pre-test probability [15]. Fig 1 shows the pre-test Probability of 47% and the post-test probability of 77% with Fagan’s nomogram. The probability of progression to Type 1 ROP is quite high. Thus treatment is required to prevent progression to Type 1 ROP.

The calculation of the risk score is based on data from a previously published cohort of infants. All these infants had signs of ROP [3]; therefore, our model is only applicable to detect those infants who were diagnosed to have ROP Type 2 and were at risk to develop Type 1 ROP. More studies, involving larger groups of infants, are needed to determine whether our model can predict all stages of ROP. Additional studies are also needed to evaluate whether our model can predict the progression of ROP in infants with signs of ROP detected with a telecamera.

**Fig 2:**
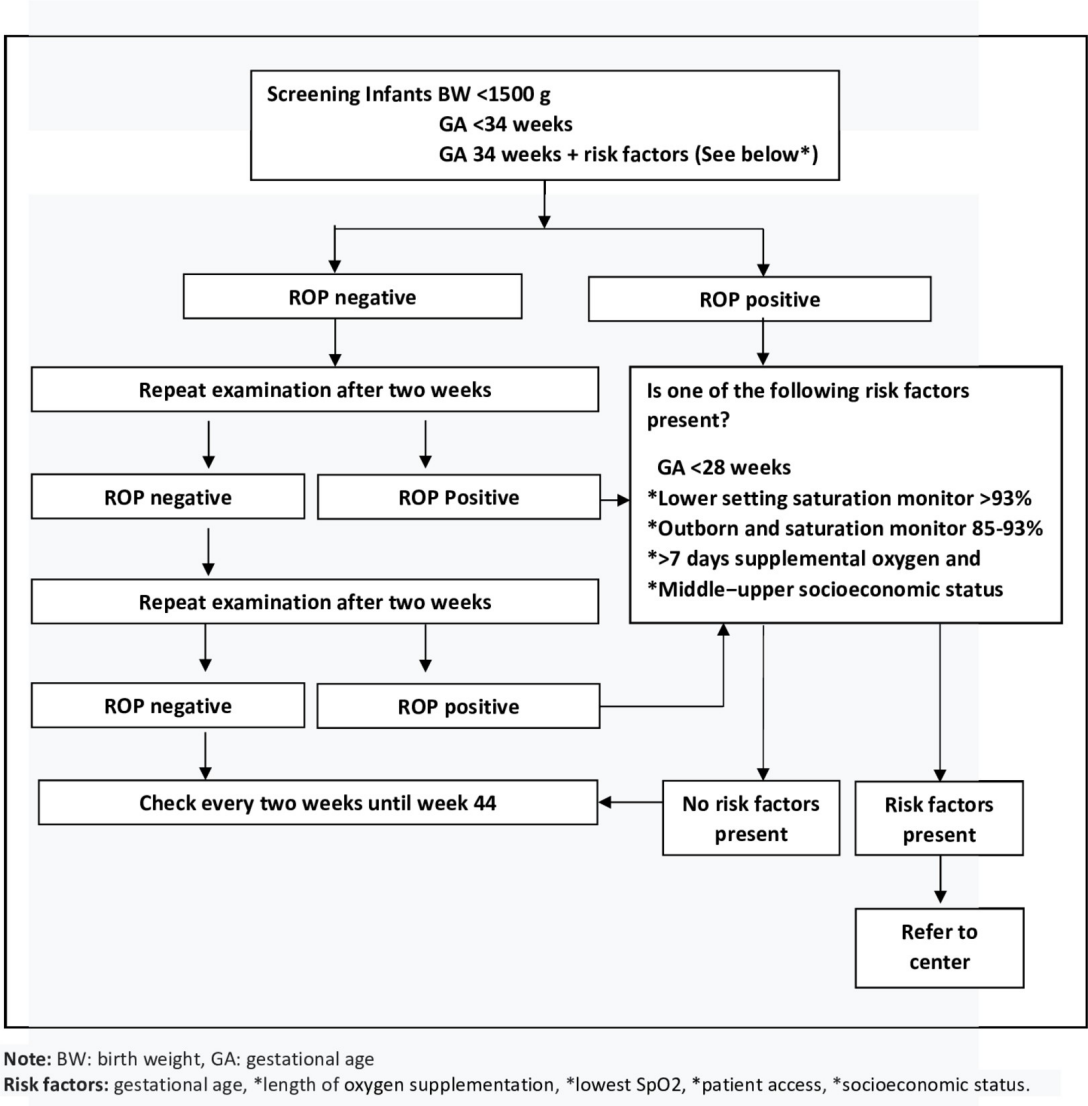
Flow chart for ophthalmologists or assisting physicians to assess the progression of ROP.

We recommend that our ROP risk scoring model be applied in neonatal care units serving high-risk infants in Indonesia and other LMICs. This model is especially suited for units that are not equipped with pediatric ophthalmologists’ resources to detect those infants who might be at risk to develop Type 1 ROP. To help the use of this model in daily clinical practice, we developed a flow chart for ophthalmologists or assistant physicians to assess the progression of ROP (Fig 2).

### Research limitation

This cohort study contains a selection bias. Only infants who had been diagnosed with ROP by ophthalmologists were included. Therefore, the probability of developing Type 1 ROP was greater than the likelihood of preterm infants’ total population. A new study involving a large group of infants at risk to develop ROP irrespective of the stage is needed to confirm the reliability of our model for detecting all stages of ROP as well as the progression of ROP to Type 1 ROP.

## Conclusion

We designed and validated a scoring model to detect those infants in Indonesia who are at risk to progress to Type 1 ROP. This model can be used in circumstances where trained ophthalmologists are not always available. A new study is needed that includes a large group of infants at risk of developing ROP.
